# Identifying gaps in technology-based intervention prevention programs and ways to foster learning and engagement with parents using serious games

**DOI:** 10.3389/fpubh.2025.1525568

**Published:** 2025-06-18

**Authors:** Michelle F. Wright, Sandra T. Azar

**Affiliations:** ^1^Department of Psychology, Indiana State University, Terre Haut, IN, United States; ^2^Department of Psychology, Pennsylvania State University, University Park, PA, United States

**Keywords:** injury, prevention, technology, serious game, home safety

## Abstract

Some researchers have implemented technologies to help overcome the barriers to reducing childhood injuries. Many of these technology-based injury prevention programs rely on individually tailored, written feedback to help improve parents’ knowledge of home safety. Serious game technologies might further aid in developing injury prevention programs that are adaptive to the unique characteristics of parents. The purpose of this paper is to review these early efforts and propose serious game technology as a critical future direction of injury prevention programs with parents. The paper begins by discussing the barriers associated with reducing childhood injuries and engaging in injury prevention programs. Studies on injury prevention programs using technology to teach parents injury prevention skills are then described. Serious game technologies are proposed as having the potential to reduce injuries and barriers. The paper concludes with describing *Home Safety Hero*, an injury prevention program, and preliminary data from parents’ game play.

## Introduction

As a significant public health issue, children’s unintentional injuries are a leading cause of children’s death and disability (1–3). In the United States, 10% of children between the ages of 2 and 6 experience an injury necessitating medical attention, with children from low-income families at an elevated risk of injury (2, 4, 5). Undoubtedly, inadequate parental supervision is a primary concern in child neglect cases (6–9). It has been argued by researchers in this field that parents’ cultivation of a safe home environment and engaging in high-quality supervision are crucial for preventing child injuries (10–14). Indeed, as much as half of child injury deaths (43%) are attributable to inadequate parental supervision (15).

Programs are needed to prevent childhood injuries that target parents, but these programs have run into barriers. For example, injury prevention programs have high attrition rates (16). Barriers to parents’ involvement in these programs including parents feeling judged and stigmatized for needing help, holding unrealistic expectations of others’ ability to help, and the hierarchical nature of parenting work where parents may feel they lose their sense of being “experts” regarding their own children (17, 18). Serious game technology has the potential to reduce these barriers by allowing scaffolded learning (e.g., building on learners’ experience and knowledge by adding support to enhance learning and mastery of tasks) and independence (19). However, to date efforts to apply serious game technology to injury prevention have involved changing the behaviors of older children not parents, usually in the context of pediatrician and fire safety (20–23).

## The purpose of this review

The purpose of this paper is to propose serious game technology as a future direction of injury prevention programs with parents, including those with cognitive challenges and those without. Such technologies have the potential to overcome barriers to reducing childhood injuries. The first section of this paper outlines barriers to reducing childhood injuries, barriers to using parenting program services for injury prevention, and how serious game technology might reduce these barriers. It describes studies on programs using technology to teach parents injury prevention skills and what is known regarding their efficacy. An explanation of how serious game technologies might enhance these programs and improve engagement is also provided.

To identify relevant literature on this topic, a comprehensive search strategy was employed across multiple interdisciplinary and subject-specific databases. These databases included PsycINFO, which covers psychology and behavioral sciences and provides access to empirical studies and theoretical discussions; PubMed, which includes research on health-related interventions relevant to childhood development and intervention programs; and ERIC, which focuses on educational research, including the use of serious games in teaching and intervention settings. Additionally, Scopus was searched to cover social sciences, psychology, education, and health disciplines, while Web of Science provided access to high-impact research in multidisciplinary fields. IEEE Xplore was also used to include technical and engineering research relevant to the development of serious game interventions for home safety.

A combination of keywords and Boolean operators was used to refine the search results. The search terms included serious games, game-based learning, digital interventions, and gamification, along with intervention-related terms such as childhood intervention, parent training, early intervention, and behavioral intervention. Parent-focused terms, including parent education, parent engagement, and parenting programs, were also incorporated into the search. Boolean operators were used to effectively combine these terms. For example, search strings included combinations such as “serious games” OR “game-based learning” OR “gamification” AND “childhood intervention” OR “early intervention” OR “parent training.” Another example included “digital interventions” OR “serious games” AND “parent engagement” OR “parenting programs”.

To ensure relevance and quality, several inclusion criteria were applied. The search was limited to peer-reviewed journal articles, conference proceedings, and systematic reviews. Only studies published between 1988 and 2023 were considered to capture recent advancements in the field. Articles were required to be in English and focus on parents as primary participants in intervention programs. The intervention focus had to include serious games specifically designed for childhood intervention programs involving parents. Studies assessing the effectiveness, engagement, or feasibility of serious games in parent-focused interventions were prioritized.

Exclusion criteria eliminated studies that focused solely on children without parent involvement, traditional non-digital interventions, opinion pieces, editorials, non-empirical articles, and articles unavailable in full text or behind restricted access. Research on serious games used for general education without a focus on intervention was also excluded.

**Figure 1:**
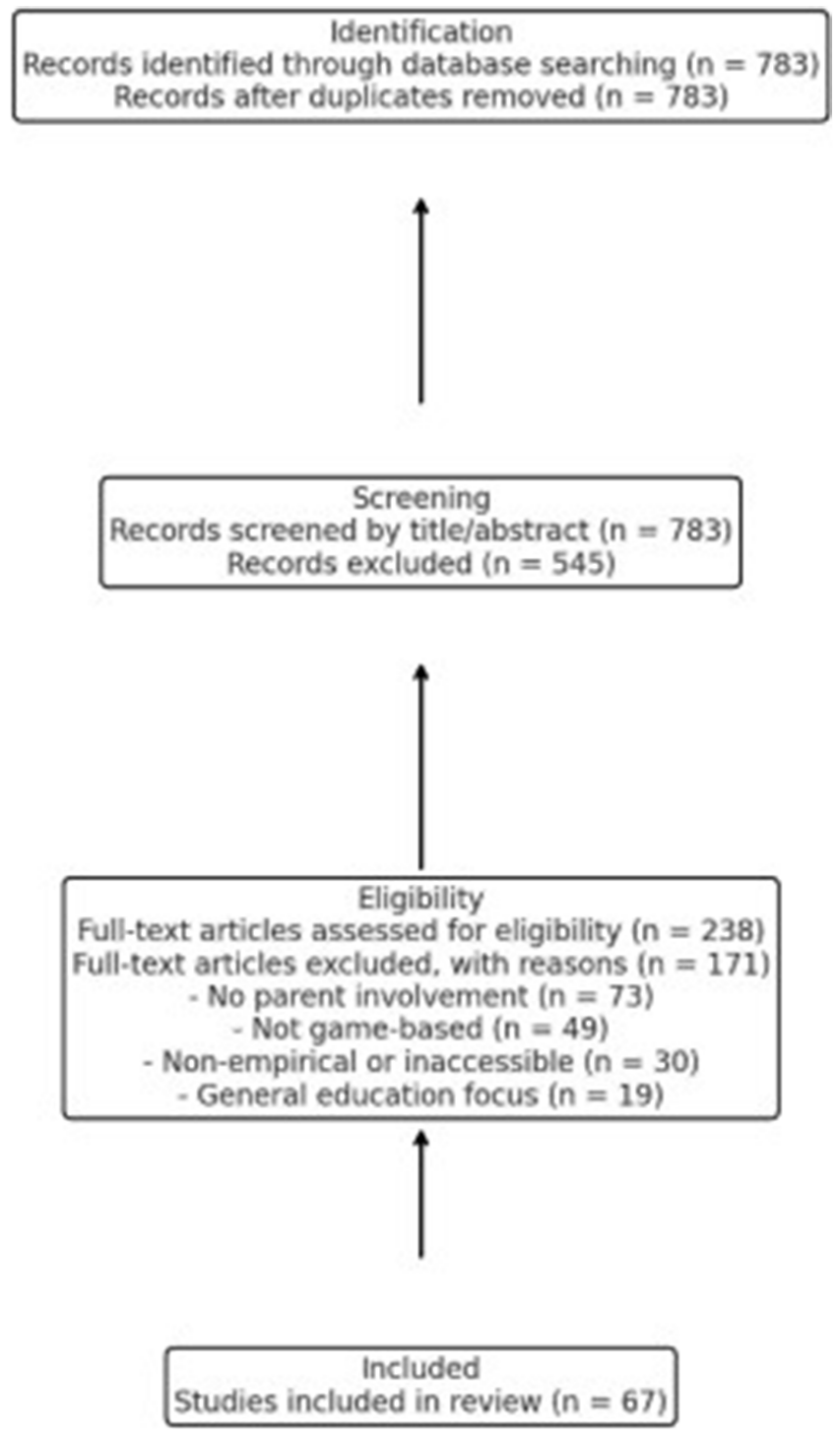
Flowchart of identification, screening, eligibility, and included studies.

The initial database search identified 783 articles. After title and abstract screening, 545 articles were excluded for not meeting the inclusion criteria. Common reasons for exclusion included lack of focus on serious games, no parent involvement, or the use of traditional rather than digital methods. A full-text review was conducted on 238 articles, resulting in 67 studies that met all criteria and were included in this review. A PRISMA-style flowchart (Figure 1) has been added to visually represent the search and selection process. The selected 67 sources were categorized into thematic areas such as game design, intervention outcomes, and parental engagement. These themes were used to organize the literature review and provide an analytical synthesis of current findings in the field.

The second section of the paper provides a detailed description of the newly developed injury prevention game, outlining its core objectives, design elements, and intended impact on parental awareness and child safety. This section also presents preliminary data collected from a small group of parents who participated in gameplay, highlighting their levels of engagement, interaction patterns, and responsiveness to various game features. Additionally, initial impressions from parents are examined to assess the game’s usability, effectiveness, and potential areas for improvement. These insights offer valuable perspectives on how digital interventions can enhance parental involvement in injury prevention strategies.

## Obstacles for reducing childhood injuries via parent interventions

Based on research with parents who show problems in supervising their children (i.e., ones who have neglect histories), parents’ cognitive challenges present barriers to reducing childhood injuries. Poor working memory, poor attention to situational cues, cognitive inflexibility (e.g., poor set shifting when encountering new information), and poor problem solving have been identified in such parents (24, 25). Slow processing speed that results from such challenges could interfere with learning using the traditional methods in parenting education. Other cognitive biases that interfere with forming a therapeutic alliance with professionals may further be detrimental to engagement in provision of such services. For example, neglectful parents have been shown to have negative appraisal styles regarding adults in their lives and a tendency to see children has having more agency in negative behaviors like purposefully engaging in injury risk behaviors (24). They also show a greater tendency to see injuries being due to luck and not their own actions (locus of control). Misappraisals of risk, perceiving injuries as the result of fate or luck, believing that injuries cannot be prevented, and failure to consider developmental changes in supervision, and such misappraisals influence injury risk and decrease the likelihood of anticipating risks (26).

### Obstacles more general to parenting programs

Parents report experiencing practical difficulties with transportation, childcare, and time constraints that hinder their participation in parenting programs. They also experience psychological barriers. Psychological barriers might include parents feeling judged and stigmatized for seeking help for basic caregiving, concerns about their confidence with basic caregiving, being labeled, and worries about confidentiality and anonymity (27). Furthermore, neglectful parents who have a high incidence of supervision related problems also have been shown to have feelings of low efficacy for their parenting abilities (28), another potential hinderance to their involvement in parenting programs. Some of this may be due to the inherent hierarchical nature of parenting work that situates parents in a lower position than social workers and others providing services (17, 18). Parents might have anxiety and/or feel unwelcomed due to class, race, or cultural backgrounds differences, resulting in them being less likely to seek help or continue with parenting programs or services (29).

Some parents have rigid and unrealistic expectations of others’ behaviors, potentially influencing their beliefs about the type of help, how much help, and whether social workers can provide help (30). In their study, Ribner and Knei-Paz found that low-income parents reported that they had many unmet needs and recurring disappointments following interactions with social workers. Such reactions may detract from parents’ motivation, increase skepticism and misconceptions about the availability, benefits, and positive outcomes of services, and once they drop out, it may reduce the likelihood of utilizing services in the future when offered to them (27).

Many parenting injury prevention programs present material didactically and require some level of literacy skills and cognitive capacities above the level of many low-income parents, especially those at risk for neglect (25). Efforts to improve engagement that reduce these obstacles are needed (17, 18). Effective parenting programs for parents with cognitive challenges involve the use of visual materials, modeling, and role playing; consistent and frequent feedback is also a necessity of these programs. Furthermore, it is important for effective parenting programs to be disseminated in home for better generalization and should involve one-on-one intervention. Oneon-one intervention is important because it tailors and pace the program to meet parents’ needs and enhance positive reinforcement. Few programs implement such strategies into injury prevention. Furthermore, staff disseminating parenting programs often lack training in injury prevention and how to present materials tailored to these adults (31–33). Nonetheless, there have been efforts to utilize technology to optimize injury prevention programs. We review these programs next and suggest future directions for injury prevention programs.

## Overview of technology-based injury prevention interventions

A handful of studies have been conducted on the use of serious game-based injury prevention interventions aimed at teaching parents about various safety risks for young children, including bicycle injuries, firearms, car seats, hot water temperature, dog bites, poison, drowning, burns, falling, and choking (34–36). These programs deliver content in multiple ways: via computer software installed using a CD-ROM on a personal computer or laptop, through a remotely hosted internet website, or mobile phone application, as well as a combination of these methods.

### Computer software

The use of computer software to deliver injury prevention interventions allows for the tailoring of curriculum to fit different literacy abilities and skills, as well as promotes efficiency of delivering the intervention to many parents in a short period of time. Many of the computer software-based injury prevention interventions create a tailored report on parents’ knowledge before the intervention, with the report identifying gaps in knowledge and providing materials to improve knowledge, and then measure knowledge of safety after the intervention.

Gielen et al. (34) conducted a randomized controlled trial of the Safety in Seconds program. Parents (*N* = 759) were recruited from emergency waiting rooms and were eligible if they had a child between four and five and half years old who was being seen or had a sibling who was being seen for an injury or a medical complaint. Parents from the intervention group (*n* = 384) completed a baseline assessment instrument on the computer, and a personalized report with tailored, stage-based safety messages was printed for them to take home and review.

An algorithm within the software utilized parents’ responses to generate a behavioral profile, and safety messages were distributed to parents based on their safety practices, with the intention of expanding parents’ knowledge of child safety. Reports for parents who scored at the highest level included a congratulation and reinforced their attention to child safety. The parents from the control group (*n* = 375) completed an assessment with content unrelated to their behavioral practices, and then they received the same guidelines as the intervention group, without tailoring of the reports. Two weeks later, parents were asked about their knowledge of child safety seat practices, smoke alarms, and poison storage knowledge, as well as if they read the report and discussed it with others. Parents in the intervention group had higher knowledge of smoke alarms, poison control, child safety seat use, and total safety knowledge scores after the two week follow-up when compared to parents from the control group.

Using a similar design as Gielen et al.’s (34) study, the *Baby, Be Safe* program was examined by Nansel et al. (35); they investigated home and car safety behaviors of parents with children (*n* = 213) ages six months through 20 months. An assessment provided tailored information based on home and car safety knowledge prior to the intervention, which involved receiving information about ways to improve parents’ knowledge based on their current knowledge or receiving generic information about home and car safety. A follow-up of knowledge gained after the intervention indicated that parents adopted home and car safety behaviors when compared to parents who received generic information. Also, when tailored information was discussed with their child’s pediatrician greater changes in home and car safety behaviors were found than when the report was not shared with children’s pediatrician.

Thus far, the studies reviewed focused on car safety, smoke alarms, and poison control. Nansel et al. (37) not only focused on these safety risks but also examined burns, falls, choking, and drowning knowledge. They administered an assessment to parents to determine their knowledge of these various safety behaviors and risks, and then either tailor safety messages to parents’ knowledge or delivered information more generally about injury prevention. Parents who received the tailored messages had significantly more knowledge of motor vehicle injuries, burns, falls, poisoning, choking, and drowning one month later when compared to parents who received generic information about injuries.

Focusing on increasing parents’ knowledge of dog bite safety, Shields et al. (36) asked 901 parents of young children to complete an assessment on dog bite safety or other safety behaviors. After completing the assessment, parents received a report, with some receiving additional information about dog bite prevention and others receiving information about other safety behaviors, such as child safety seat, smoke alarms, and poison storage knowledge and behaviors. The findings from this research revealed that parents’ knowledge of dog bite safety increased when they received the specific safety information about dog bites. Shields et al. (38) utilized a similar design for increasing parents’ knowledge of child safety seats, smoke alarm use, and safe poison storage. In addition to collecting data on parents’ self-reported knowledge of these safety concerns, Shields et al. also conducted in-home observations. Their findings suggested that receiving tailored safety information increased parents’ knowledge of home safety information, but discrepancies were found between observed and self-reported behavior. Observed home safety behaviors were lower than self-reported home safety behaviors.

Overall, the findings suggest getting tailored reports was more effective than receiving general information. These studies presumed that parents would read and understand the content of the reports. Some safeguards with respect to literacy were noted (e.g., reports generated with reading level considered) but parents might continue to have difficulty with reading comprehension, which might hinder their ability to implement child safety. Computer-generated, tailored reports could be enhanced through the incorporation of some serious gaming techniques, such as having auditory versions of written materials. Furthermore, because tailoring reports was effective for the safety prevention programs reviewed in this section, serious game technologies could also tailor the feedback provided to parents. Presenting information in an auditory format might improve parents’ comprehension of safety-related information, resulting in better understanding of home safety knowledge (39). These auditory materials might also include various examples of how parents can keep their children safe. Furthermore, reading and understanding materials from the reports involve passive learning that might be best augmented from implementing more engaging content involving video and interactive lessons, storylines, and feedback (40). The use of serious game techniques involving interactive and visual contents might help to increase parents’ engagement in the injury prevention intervention and their implementation of safety-related behaviors in their homes. Parents who are better able to immerse themselves into the learning experience through interactive lessons and storylines might have better recall and implement the information they are learning (39).

### Internet website

The use of the internet for delivery of injury prevention has the potential to reach more people and provides interactivity and cheaper access to materials than inperson contact or telephone calls. Following the many enhancements to the internet, such as Web 2.0, childhood injury prevention researchers have developed programs to promote home safety awareness and knowledge through websites. The use of the internet to deliver home safety programs enhances and provides unique experiences to parents based on their needs and represents an improvement to injury prevention programs that utilize tailored reports.

*MyHealthyChild* is a web-based intervention developed by Christakis et al. (41). The website includes various topics (e.g., smoke detector use and testing, car seat use and installation, hot water temperature, bicycle helmets safety, firearm storage, sudden infant death syndrome), with the content tailored to parents’ responses to questions about their child’s age and other information (e.g., status as smokers, family income). For example, if one parent smokes, then they were presented with safety information related to smoke detector use and testing. Parents were randomly assigned to one of four groups: (1) parents who received access to *MyHealthyChild*, where they received web content, read topics in which they were interested, and received notifications from their child’s pediatrician, (2) parents who received provider notifications from their child’s pediatrician only, (3) parents who received access to MyHealthyChild only and did not receive notifications from their child’s pediatrician, and (4) parents who received usual care, had no access to the web content, and visited providers as usually, without notifications from their child’s pediatrician. Parents completed baseline questionnaires and then were involved in one of the four groups three to 14 working days prior to their well child visit. Two to four weeks after the well-child visit, parents participated in a telephone interview about the topics discussed at the visit and their preventive practices for each of the topics covered by the *MyHealthyChild* website, regardless of whether the parent accessed the website. Findings revealed that parents in the *MyHealthyChild* and notification group and in the notification-only group discussed more *MyHealthyChild* topics with their child’s pediatrician. Increased implementation of safety recommendations was reported by parents in the *MyHealthyChild* and notification group as well as the *MyHealthyChild* and no notification group.

Van Beelen et al. (42, 43) evaluated the efficacy of the *E-Health4Uth Home Safety* program using a sample of 1,292 parents with an 11-month-old child. Parents were randomly assigned to either the *E-Health4Uth Home Safety* module, a web-based, tailored safety advice mobile condition, and discussion at the well-baby visit (*n* = 696; treatment condition) or provided with written safety information leaflet at the well-baby visit (*n* = 687; control condition). The *E-Health4Uth Home Safety* module delivers safety information concerning the prevention of falls, poisoning, drowning, and burns. Prior to completing the module, parents completed a safety assessment questionnaire, with parents’ answers being used to tailor safety advice that parents could immediately read online. The information was also tailored to their child’s name, current situation, and safety behavior. After reading the information, parents developed an implementation-intention plan that specified what, when, and where to improve their safety behavior and how they would implement safety in their home. The tailored safety advice was also emailed to the child health care professional to discuss at the well-child visit. Before and one-month after the intervention or receiving the leaflet, parents completed a follow up self-reported questionnaire about their specific child safety behaviors for the prevention of falls, poisoning, drowning, and burns. Parents in the intervention condition showed significantly less unsafe behavior for falls, poisons, burns, and drowning when compared to parents in the control condition.

Both the *MyHealthyChild* and *E-Health4Uth Home Safety* programs demonstrate efficacy for helping to promote awareness and increase parents’ knowledge of childhood injuries. The websites were developed to provide parents with access to curriculum on childhood injuries and how to implement safety precautions to reduce such injuries. Parents’ ability to learn about home safety might improve through the inclusion of videos, quizzes, discussion boards, and other interactive activities through an internet-based injury prevention program. Including additional interactive activities might increase parents’ engagement, which might subsequently increase their knowledge of home safety and ability to resolve safety issues within their homes. Similar to the use of tailored reports of safety behaviors and recommendations, these internet website-based programs rely on parents’ ability to read and comprehend safety information, something that might be challenging for parents with cognitive challenges. Including visual materials and scaffolding of materials might increase parents’ learning of home safety (39). It is unclear how these tailored reports might improve home safety knowledge among parents with cognitive challenges who might need more interactivity to promote learning.

### Other technology-based methods

Researchers have also utilized other types of technologies, specifically mobile applications, as well as a combination of different methods (e.g., internet website and mobile application) to help parents increase their knowledge of injury prevention (44, 45). The research reviewed in this section involve one proposal and one efficacy pilot study for how the researchers would implement and evaluate injury prevention programs involving mobile applications and combinations of other technologies.

In their proposed study, Chow et al. (45) describe an injury prevention program involving the use of both internet website and mobile application to enhance parents’ knowledge of home safety. The website provides informational, educational, and motivational support to parents through providing content on safety information and online games to deliver safety messages. In conjunction with the website, parents will also have access to a mobile application to improve parents’ knowledge of child-related injury prevention in the home. The content of the website and mobile application are the same. Other than the function as a knowledge hub, the website and the mobile application include online consultations, a discussion board, interactive games, and video demonstrations to improve parents’ knowledge, attitudes, intentions, and perceived behavioral control toward home safety practices. Chow and colleagues plan to provide parents with home safety counseling on falls, scalds, sleeping safety, suffocation, drowning, poisoning, toy hazards (choking), concussion, sunburn, and driving via the website and mobile application. Prior to the intervention, parents will complete self-reported questionnaires on their home safety knowledge, attitudes, intentions, perceived behavioral control, and actual behaviors. After completing questionnaires, parents will either receive an information parenting booklet on home safety or receive the booklet plus receive access to the website and mobile application. Questionnaires will be administered again to parents at 2, 6, 9, 12, and 18 months.

Applying gamification techniques to a mobile application-based injury prevention program, Burgess et al. (44) developed *Cool Runnings*, a mobile-application game designed to increase parents’ knowledge about the causes of burns and first aid for burns/scalds. The design of this study involved comparing a control group with an intervention group. The 6 month intervention involved participants receiving 9 intervention messages via the application. The messages were related to risks of hot beverage scalds, risks of developmental stage-based burns, and burn first-aid treatment. The messages were delivered through infographics, 30s videos, and motion graphics over 3-week intervals. Participants were able to engage with the application through activities such as answering pop quizzes and completing missions (e.g., uploading pictures to the application) that reinforced each of the intervention message themes. They received points for completing quizzes and missions, and these points were displayed on a weekly leaderboard in the application; points could be redeemed for rewards (e.g., shopping and movie vouchers). The control group also utilized the application interface but with only the infographics, and they did not engage with the material through pop quizzes, missions, points, or the leaderboard. Knowledge about first aid treatment and the main causes of burns or scalds were accessed prior to the intervention and six months after implementation. The intervention group had significant improvements in overall knowledge at the post-test than the control group. Gamification techniques were positively correlated with knowledge change.

Burgess et al.’s (44) study on the efficacy of a mobile application and Chow et al.’s (45) proposal for creating a mobile application involved utilizing various technology-based learning devices, such as the inclusion of visual and auditory information. Furthermore, Chow et al. (45) described the process to record progress in the game and correlate such progress to self-reported questionnaires on supervision and home safety. It was unclear from Burgess et al.’s (44) description of their study whether they recorded and utilized any of the data gathered from their mobile application, and whether the messages sent to participants were read or received. Thus, the possibility to utilize data generated from mobile-based applications or other technology-based interventions and gain a meaningful understanding of how such data might help inform intervention outcomes is uncertain. Techniques to scaffold materials, involve multiple trials, and include immersive storylines are needed for improving engagement and learning (39, 40). Adaptability of the materials to address literacy needs were not described, something that might be especially important for parents with cognitive challenges.

## What’s next with technology-based injury prevention?

This section outlines the existing research and application of serious game technology as a next step for injury prevention programs. This section concludes with a description of our serious game, *Home Safety Hero*, and preliminary data from parents’ game play, their engagement with the game, and their initial impression of the game.

### Serious game technology

Developed in the late 1990s, serious games are defined as digital games used for purposes other than entertainment (46). Such games have applications to education, industry, engineering, military, and medicine (47). Not only are serious games effective, such games foster continuous learning experiences, the ability to impart skills, knowledge, and attitude, and provide new opportunities for collective learning and training while also incorporating fun elements to engage learners (48–50). Such technology has the potential to increase engagement and reduce attrition by promoting autonomy (19). The ability of serious games to address engagement issues associated with knowledge acquisition is important and such games have the potential to incorporate fun factors to immerse learners in an active and dynamic learning environment (51). Serious games are able to push learners to complete and overcome challenges through providing immediate feedback. Educational objectives are combined with specific evidence-based game mechanics to support learning and generalization of skills learned through serious games. To our knowledge and after a thorough review of the literature, serious game technologies have not yet been applied to injury prevention programs with parents.

Serious game technology might address some potential challenges for learning about injury prevention, including the ability to reach the new generation of parents who have grown up in a fully digitalized society and the need to provide a more engaging and motivating way of instilling skills and knowledge that can be utilized in the real world. This technology might also be a solution for adapting parenting programs to meet the needs of cognitively-challenged parents. Effective learning requires addressing individual differences in parents’ cognitive abilities and rate of learning new skills (52). To do this, serious game technology can include injury prevention programs with individually tailored content and feedback and allow for the presentation of visual materials to fit special learning needs. The ability to scaffold materials might increase mastery and reduce failures, as well as allow almost infinite number of practice trials to strengthen learning (39). Difficulties with literacy can be avoided through the inclusion of visual and auditory information in the game. Serious games also increase the reach of injury prevention programs. Learning can also be enhanced through intrinsic motivation to engage parents with difficult learning content (53). Situated learning is possible in these games, allowing for the incorporation of storylines, long-term learning goals, and gradual increase in level difficulty (40).

It might be particularly hard for parents to receive feedback from parenting program staff about their ability to mitigate child safety injuries, as parenting is often conceptualized as a “natural process” and admitting to needing help might be embarrassing and highly stigmatized. The use of games might make receiving such feedback easier for parents by providing immediate, tailored feedback in a private setting. Because parents can work independently, it is possible for them to establish autonomy and self-efficacy regarding their ability to identify and remove dangers in their home environment. Attrition rates might also diminish because the stigma associated with needing help or not knowing something is removed. The storyline provides a context for learning and helps to increase parents’ engagement with the game. It is also possible to track progress through the game and increase or decrease challenges when ready or needed (54). Program staff can utilize parents’ progress to praise knowledge learned and provide opportunities for growth. Next, we describe our immersive serious game, Home Safety Hero, and present some preliminary data from a small sample of mothers with young children.

### Home Safety Hero

Home Safety Hero was programmed using Unity 3D and it provides a simulated home environment designed to increase skills in identifying and resolving home safety hazards. The game was designed with elements from previous injury prevention work (55, 56) to maximize skills and increase engagement with intervention (19, 57). Visual and auditory prompts are provided throughout the game to increase accessibility for players of various literacy levels. Instructions and praise are presented in written form and voiceover. Encouragement is given via voiceover and celebratory graphics (e.g., confetti after completing levels). All hazards in the game have sound effects to promote investment and engagement. Hazards are distributed within rooms in a simulated home, including the kitchen, bathroom, bedroom, living room, and hallway (see Figure 1 for examples of the rooms). Players navigate through the rooms from a first-person perspective by using the keyboard and mouse. Hazards included in the game were reported as the most commonly occurring injuries in preschool-aged children presenting at the emergency room (2), and include burns (e.g., lit candles), drowning (e.g., bathtub full of water), choking/suffocation (e.g., plastic bag), poisoning (e.g., bug spray), falls (e.g., toys in the walkway), weapons (e.g., pistol) and sharps (e.g., knives). To help with learning to identify hazards, a symbol (i.e., a yellow triangle) appears above each hazard when the player approaches the hazard for the first time. Each level has a timer and players must identify or resolve hazards before the timer runs out. If the timer runs out, players must repeat the level; however, no punitive language is provided when levels are repeated and instead encouragement is provided players cannot progress to the next level until all hazards are identified or resolved.

**Figure 2:**
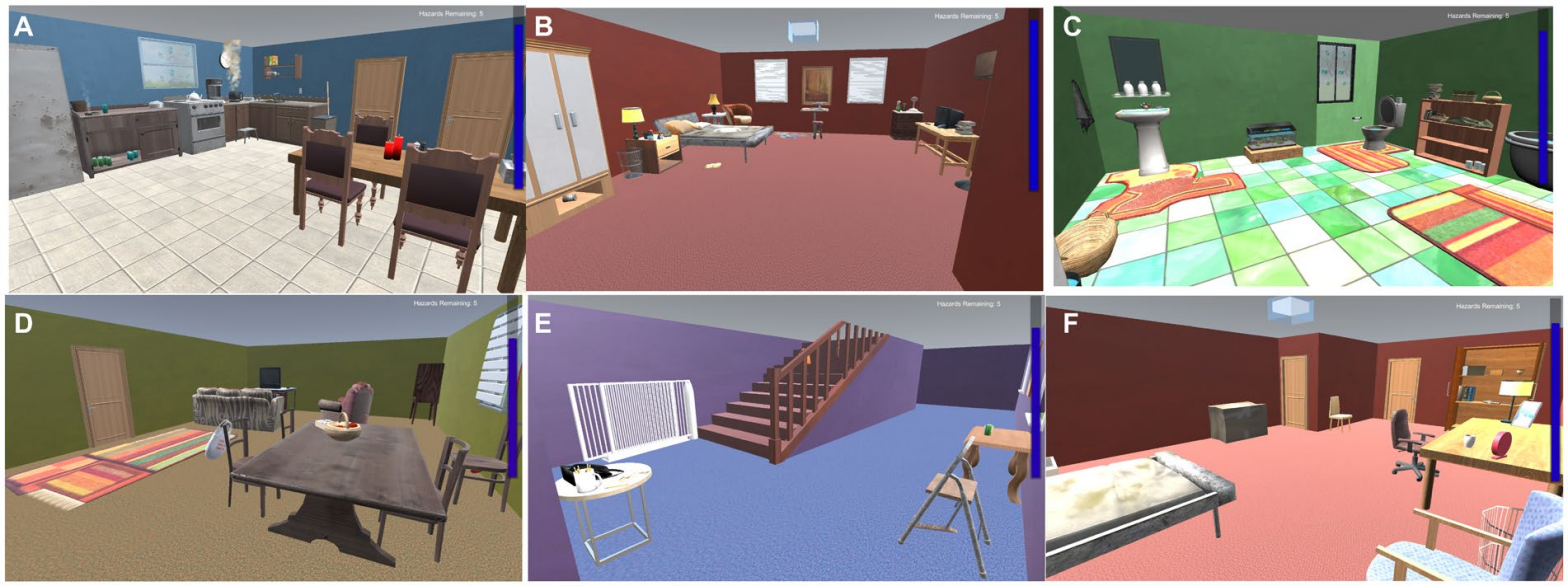
Images of the six room designs in *Home Safety Hero*. **(A)** Level 1; **(B)** Level 2; **(C)** Level 3; **(D)** Level 4; **(E)** Level 5; **(F)** Level 6.

**Figure 3:**
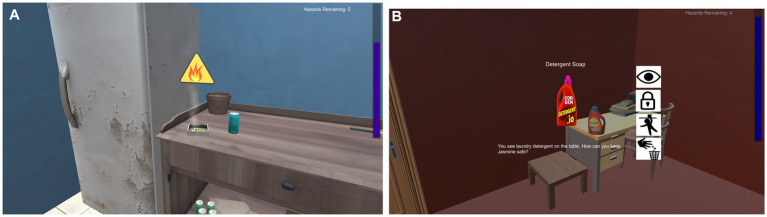
Images of game play for identification resolution phases. **(A)** Image of game play from identification phase showing the yellow triangle hint for the burning cigarette hazard. The time bar is on the right. **(B)** Image of game play from resolution phase after selecting the detergent soap hazard. Icons for what should be done to resolve the hazard are listed on the right. The text reads, “Detergent Soap” and “You see laundry detergent on the table. How can you keep Jasmine safe?”.

There are two phases of *Home Safety Hero*, including Identification (e.g., the task is to locate and click hazards when encountered; 30 levels) and Resolution (e.g., the task is to identify and then pick a solution for resolving/removing the hazard; 12 levels; see Figure 2 for scenes from the game). In the Identification Phase, level difficulty increases by increasing the number of hazards in each level and by combining different hazard types (e.g., fire hazards with drowning hazards; mixed levels) as the levels progress. They played levels with single hazard types presented first and must identify hazards in rooms with five hazards, followed by seven hazards and then 10 hazards. Next, they play mixed levels, which also increase in difficulty from our hazards to six hazards then to eight hazards and ten hazards. In the Resolution phase, level difficulty increases by including hazards with more difficulty solutions as the levels progress. For all phases, players are presented with a storyline that monsters have hidden hazards in a virtual home environment and that they need to keep hypothetical children safe by going through rooms (i.e., kitchen, bathroom, hallway, living room, two bedrooms) to remove and/or resolve hazards. The game records the total time to complete each level, reaction time for finding or resolving hazards, and total amount of clicks for finding hazards. More specific details about the game are reviewed elsewhere (58) (see Figure 3).

### Preliminary data

In this section, we present some of the data we collected from parents on their game play, their engagement with the game, and their initial impression of the game. We recruited eight mothers (*M* age = 32.17 years) from a local parenting program in central Pennsylvania to play *Home Safety Hero* and answer questionnaires about the game. They were introduced to the study using an announcement during an enrichment program and given flyers about the study’s purpose and how they could participate if they were interested (e.g., calling the research lab and scheduling an interview). Approximately 10 flyers were distributed, and eight mothers called the research lab to express their interest in the study. All consent to participate in the study. Mothers had 12.83 years of education (*SD* = 2.98 years) and most of the mothers (66%) identified as white. They played *Home Safety Hero*, and then answered questionnaires about their perceived engagement with the Identification and Resolution Phases, separately, using the Perceived Usability subscale of the *User Experience Survey* [items rated on a scale of 1 to 5, with 5 being strongly agree; O’Brien and Toms (59)]. An example item included: I think I would play this game frequently. Mothers also rated how much they learned from the game (on a scale of 1 to 10, with 10 indicating they learned a lot) and specified what they learned from the game using an open-ended prompt for both the Identification and Resolution Phases. The same procedure was utilized to assess how much fun they had while playing and they specified what elements made the game fun, as well as indicated how much they were involved in the game play and they specified what elements they felt involved them in the game. Lastly, mothers responded to two open-ended questions regarding anything they learned from the game and what features of the game they enjoyed.

Many home safety programs focus on improving hazard identification and resolution (e.g., 60), and reaction times were used as a proxy for measuring how effective learning was, with quicker reaction times indicating better learning. Reaction items were calculated by averaging the total time to identify or resolve hazards for the Identification and Resolution phases, while accounting for the total number of hazards per level. Mothers took on average 13.36 (*SD* = 3.47) seconds to identify hazards in the single hazard levels with five hazards, 6.40 (*SD* = 2.07) seconds in the single hazards with seven hazard levels, and 4.78 (*SD* = 2.04) seconds in the single hazards with ten hazards levels. Similar patterns were found for the mixed levels such that mothers took on average 6.27 (*SD* = 2.01) seconds for four hazards, 6.04 (*SD* = 2.32) seconds for six hazards, 5.82 (*SD* = 2.27) seconds for eight hazards, and 4.53 (*SD* = 1.86) seconds for ten hazards. In addition, it took mothers 19.15 (*SD* = 5.35) seconds for easy solutions, followed by 19.14 (*SD* = 5.35) seconds for medium solutions, 17.56 (*SD* = 3.51) seconds for hard solutions, and 15.90 (*SD* = 4.62) seconds for expert solutions.

Mothers rated their perceived engagement for the Identification Phase as a 4.20 (*SD* = 0.36) and 4.07 (*SD* = 0.60) for Resolution Phase, indicating that they agreed to strongly agreed that the game was engaging using a 5-point rating scale. Ratings ranged from 7.08 to 8.92 (on a scale of 1 to 10) for how much they learned, how much fun they had, and how involved they felt while playing the game, indicating that mothers generally believed they learned something from the game, that they believed the game was fun, and that they felt involved with the game play in both the Identification and Resolution Phases. Comments concerning what they learned generally revealed that mothers learned about hazards they had never considered to be harmful for children, like picture frames and nail polish. Mothers believed that the different rooms in the virtual home, hazards changing location, and the realistic nature of the virtual environment made the game fun and helped keep them involved in the task of identifying and resolving hazards.

### Future directions of *Home Safety Hero*

Overall, based on mothers’ reaction times on the Identification and Resolution Phases, mothers were faster at spotting risks in the home and resolving those risks from earlier levels to latter levels, even with increasing the number hazards and increasing the difficulty of the solutions. We are still in the process of testing other elements of the game and with different populations who might benefit from our intervention. We have also added a Distraction phase since the earlier data collection with mothers. The Distraction Phase is similar to the Identification Phase, except that players identify hazards while experiencing various distractors in the home environment (e.g., hearing emergency sirens, listening to voicemails). In later levels of this phase, a child moves around the virtual room while players must identify hazards nearest to the child to progress. Reaction time and correct answers are also recorded in this phase. We are in the early planning stages for a randomized controlled trial with pregnant and parenting teens in an urban area of Pennsylvania.

## Conclusion

We have reviewed technology-based interventions aimed at parents for preventing injuries to their children in the home environment. Our conclusion is that there are few technology-based injury prevention programs, with many of these programs providing reports on current home safety knowledge and how parents can improve their knowledge by reading the information provided as part of the intervention. Although these programs have demonstrated efficacy regarding the acquisition of knowledge, parents with cognitive challenges, particularly reading comprehension difficulties, might benefit more from the auditory presentation of materials. Internet-based injury prevention programs rely on tailored content for parents based on assessment results. Similar to tailored-based reports from other injury prevention programs, internet-based programs also rely on parents’ ability to read and understand safety information, which might be challenging for parents with cognitive problems. Visual materials and scaffolding of materials might increase parents’ knowledge of child safety.

We propose serious games as a promising technology-based tool for injury prevention programs in parents’ homes. Serious games have the potential to reduce the need for professionals and extend the service reach for universal injury prevention. In addition, serious game technology might be especially useful for some parents who might have difficulty with or lack access to traditional in-person parent training. There is great potential for serious game technology to increase parents’ autonomy, reduce costs of parent training, and increase engagement with learning about child safety risks. Increased engagement is important for injury prevention programs to reduce attrition and foster parents’ awareness, knowledge, and ability to reduce injuries in their homes.

No attention has been given to the application of serious games to injury prevention interventions prior to our development of the game, *Home Safety Hero.* Preliminary data from the game with a small sample of mothers indicates that they improved on their ability to identify and resolve hazards while playing the game, and that they felt engaged with the task. Reaction time improvements demonstrate that mothers were quicker at identifying risks in the home and at taking action when encountering risks. Mothers also reported that the game helped them learn, was fun, and that they felt involved in the process of learning about home safety. We continue to further develop Home Safety Hero to increase the reach of injury prevention programs for parents, as well as evaluate the scalability of the game for use in home visiting programs.
